# True Random Number Generator (TRNG) Utilizing FM Radio Signals for Mobile and Embedded Devices in Multi-Access Edge Computing

**DOI:** 10.3390/s19194130

**Published:** 2019-09-24

**Authors:** Kyungroul Lee, Manhee Lee

**Affiliations:** 1 R&BD Center for Security and Safety Industries (SSI), Soonchunhyang University, Asan, Chungnam 31538, Korea; carpedm@sch.ac.kr; 2 Laboratory of High Performance and Secure Computing, Department of Computer Engineering, Hannam University, Daejeon 34430, Korea

**Keywords:** random number, true random number generator, FM radio signal, random number generation, cryptography, mobile communication, multi-access edge computing (MEC)

## Abstract

As transmissions of data between mobile and embedded devices in multi-access edge computing (MEC) increase, data must be protected, ensuring confidentiality and integrity. These issues are usually solved with cryptographic algorithms systems, which utilize a random number generator to create seeds and keys randomly. Their role in cryptography is so important that they need to be generated securely. In this paper, a true random number generator (TRNG) utilizing FM radio signals as a source is proposed. The proposed method can generate random numbers with high entropy, increased by at least 118% and up to 431% compared to existing generators.

## 1. Introduction

As IoT (Internet of Things) environment becomes more common, the number of mobile and embedded devices in multi-access edge computing (MEC) keeps increasing. To secure cryptography algorithm encrypt/decrypt communication between these devices, it is necessary to generate keys and random numbers for those cryptography systems. For this purpose, two types of generators are in use: pseudo-random number generators (PRNGs) and true random number generators (TRNGs). PRNGs, widely used in modern cryptography systems, generate a sequence of numbers almost at random, but are not truly random. This is because the software methods in PRNG are deterministic, and the sequence of numbers are determined by an initial value and keys. Various mobile and embedded devices in multi-access edge computing are connected to the network as IoT develops, thus keys and random numbers need to be generated to transfer information between these devices. Generating a random number from an effective random number generator is very important because a lot of communication and information is being shared between electronics. Moreover, the generated random number should not be predictable, nor should it be generated by a third party.

To cope with PRNGs’ weaknesses, studies have been carried out on TRNGs that can truly create random numbers using hardware methods [1,2,3,4,5,6]. The main topic for TRNGs has been to find sources that are random enough to safely create seeds and keys. For example, Hennebert et al. used wireless statistics and physical sensors [7,8]. Yoo et al. used the race condition in GPU (Graphics Processing Unit) [9]. Even though there were a lot more studies for better sources [10,11,12,13,14,15,16,17,18,19,20,21,22,23,24,25], they mainly considered the randomness of the source, not focusing on the noise from the source. This study’s experimental results revealed that previously proposed generators create random numbers with rather low entropy, which might result in security breaches.

Therefore, the objective of this study was to propose a TRNG that increases randomness by maximizing noise and data, rather than by using data alone as a random number as in previous generators. For this, frequency modulation (FM) radio signals have been used as sources. FM radio signals function is well because even if a signal is collected at the same time and same location, collected signals are different due to interference. In other words, it is possible to obtain truly random values from radio signals that are completely unpredictable, even in the same environment. In particular, it would be very beneficial to use FM radio signals for mobile and embedded devices, as these are usually located at various points at which FM radio signals would have unpredictable noises.

The idea is to get noisy FM signals from the antenna and to accumulate more noises when recording the sound. By tweaking parameters such as audio file formats, mixed channels, and channel change durations, a TRNG that was up to 431% better than previously proposed TRNGs was acquired. Considering that servers use various hardware modules such as lasers, the visible spectrum, super-luminescent LEDs, monitors, disks, MICs, TVs, and chaos systems for random number generation, the proposed system with a simple FM receiver module is meaningful because IoT devices can construct very good TRNGs at a relatively low cost.

The advantages of the proposed true random number generator using FM radio signals are as follows. First, there are various channels for wireless communication, such as mobile communication, DMB, and so on; nevertheless, FM broadcasting is still used worldwide, and becomes more important in disaster situations. It is expected that FM radio signals are continuously broadcasting, so using it as a TRNG is likely to be useful in the future, too. Second, FM bands receive different signals according to their geographic locations, and different signals are collected due to ambient noises even at the same location and time. This characteristic is a good source for generating true random numbers, because it is heavily affected by reception and ambient noise.

This paper is structured as follows. Section 2 introduces the related works and Section 3 introduces the proposed system. Section 4 present experimental results, and Section 5 is the conclusion of the study

## 2. Related Works and Adversary Model

### 2.1. A. Related Works

Hennebert et al. proposed a random number generator based on wireless statistics with erroneous packets, a received signal strength indicator (RSSI), and a link quality indicator (LQI) as sources [7]. In this generator, signal strength and channel quality were reflected. The advantage of this approach was that no additional hardware is necessary. According the experimental results, the LQI and erroneous packets were the most effective sources. The LQI generated random numbers with the highest entropy, and the estimated entropy was 0.47 when a sample size was 8 bits.

In another study, the same group of authors proposed a new random number generator based on physical sensors [8]. They used physical sensors, an X accelerometer, a Y accelerometer, a Z accelerometer, vibrations, and magnetism, as the sources for random numbers. The accelerometer, magnetometer, vibration sensor, and internal clock could generate high-quality random numbers. The accelerometer had a sample size of 9 bits and the entropies estimated for X, Y, and X were 0.22, 0.42, and 0.36, respectively. Moreover, random numbers collected from the vibration and magnetic sensors had sample sizes of 16 bits and estimated entropies of 0.17 and 0.62, respectively. However, sensors measuring aspects such as temperature and air pressure could not generate random numbers effectively.

Yoo et al. proposed a random number generator based on race conditions in parallel computations on GPU [9]. For improved security, they generated random numbers by calling a high-speed random number generating library functions such as cuRAND. They successfully verified that the GPU could be used as a hardware random number generator with a physical source. Entropies of random numbers with a sample size of 4 bits generated from GTX 690 and GTX 780 were measured as 0.50 and 0.60, respectively.

TRNG can be applied to various applications that use cryptographic algorithms, and is particularly effective for user authentication protocol, which is one of the important applications for security. Ding et al. proposed an efficient multi-factor user authentication protocol using WSNs (Wireless Sensor Network) [26]. User authentication protocol must securely transfer the information related to user authentication, and uses cryptographic algorithms and a secret key to ensure security. However, if the secret key is exposed or guessed, the security of the authentication protocol is not guaranteed. For this reason, a true random number can be used to enhance the security of the protocol. More specifically, it can be used as a one-time password to replace a password, long-term secret key, seed value for all types of keys, or shared secret key.

Various methods for generating random numbers have been researched, including PRNG and TRNG. PRNGs generate a random number with a long length using seed values with short lengths. These generators include a linear feedback shift register (LFSR), a linear congruential generator (LCG), and multiplication. TRNGs generate a random number using randomness based on physical aspects. For this reason, measurement of randomness is required, because randomness changes with change in the generator environment. The random number generated is classified into a software method and a hardware method based on the generation methods. The software method generates random numbers based on the information of the terminal, and the information includes external and auxiliary devices and system information. However, there is a drawback here, in that the random numbers generated in environments of the same terminals are all the same. The hardware method generates random numbers based on physical aspects using sources such as electrical and electronic circuits, chaotic signals, and systems in the natural world. This method mainly uses noises that are difficult to predict in order to get the seed value of the random number. The disadvantages are that an extra device is required, and the device is difficult to implement and attach to the system.

### 2.2. B. Adversary Model

All the related works described in our research shared a similar adversary model. Even though some researchers such as Ding Wang et al. have elaborated on adversarial models [27], we used a simple adversary model and focused on designing a TRNG. The main capabilities and limitations of an adversary (*A*) are described as follows. First, an *A* is able to collect all communications between sensor nodes. Second, an *A* can receive all FM radio signals at the same location as a target. Third, an *A* may obtain the previously generated random numbers. Fourth, an *A* may acquire all implementation details of the TRNG. Fifth, an *A* cannot stage a physical attack on the devices. With these capabilities and limitations, we assume that the *A* simply tries to predict the next random numbers that will be used for creating keys in the devices.

## 3. Proposed TRNG Model and Adversary Model

**Figure 1:**
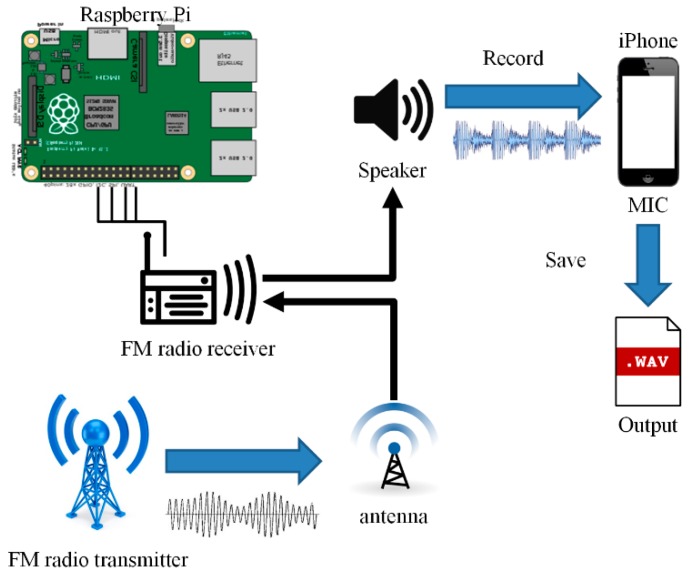
System design.

**Figure 2:**
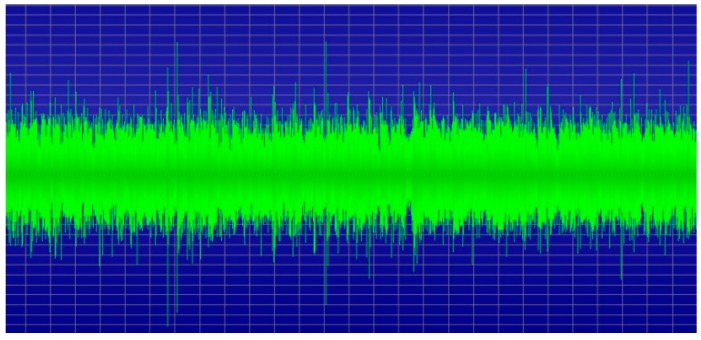
Collected analog radio signals.

To collect as many noises as possible, an FM radio signal was first received, then the signal was output directly through a speaker. The sound from the speaker was recorded and the recorded file was used as a source for the TRNG, as shown in Figure 1. An FM radio receiver with a speaker jack and an antenna jack was used. When the receiver was tuned to a channel, the FM signal sound could be heard from the speaker connected to the module. A Raspberry Pi board was used to control the FM radio receiver. The noise was mingled in all the transmission processes collected from various modules such as the FM radio transmitter, antenna, speaker, and microphone. The collected noisy analog FM radio signals, as shown in Figure 2, were transformed into an audio file.

The multiple number of different FM signals reaching a single location enhances the randomness of TRNG sources. Radio channel signals differ with respect to countries and locations, but should not be assumed that one FM receiver can catch several channels at one position. With the existence of multiple channels, two additional methods can be structured that change channels, thus proposing three methods: file format, channel, and delay.

**File format:** When recording a sound, an iPhone stores it in the M4A file format. Since the format compresses the original sound, the WAV file format is considered as having uncompressed sound information. In this study, random numbers generated by file formats such as WAV and M4A were compared.

**Channel:** It is quite a straightforward assumption that more noises are available among multiple channels than are present in a single channel. To verify this, random numbers generated from multiple channels and a single channel were compared.

**Delay:** As an additional parameter of using multiple channels, this study used delays when the channel was changed. To see how much randomness was obtained, random numbers generated from different delays were compared.

## 4. Experiment Results

### 4.1. Randomness Measurement Method

To measure the randomness of the random numbers, an entropy estimation tool provided by NIST (National Institute of Standards and Technology) was used. Recently released 800-90b estimates randomness using various estimation methods according to entropic properties [2]. Estimation methods are classified into independent identically distributed (IID) and non-independent identically distributed (non-IID). Since radio signals are produced continuously, non-IID was used for estimating randomness.

### 4.2. Randomness Estimation According to File Format

**Figure 3:**
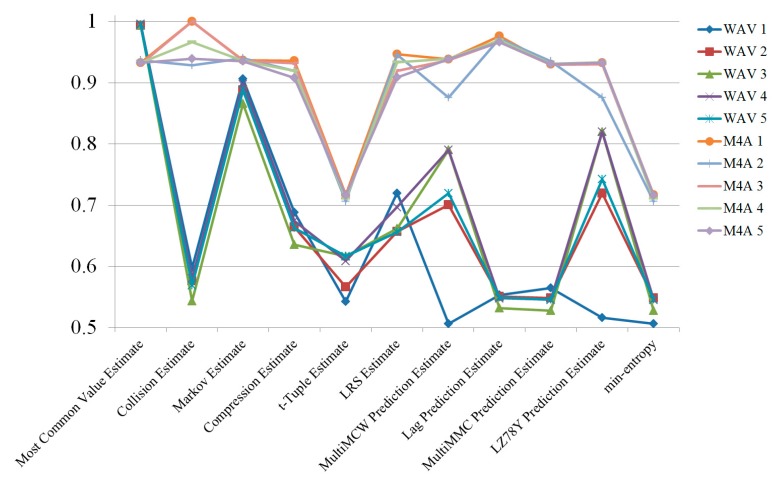
Randomness estimation results of radio signals collected from a single channel.

First, randomness was compared between M4A (compressed data) and WAV (uncompressed raw data) formats. To measure randomness, radio signals were captured five times and 10 files were made using the two formats. The randomness estimation results are shown in Figure 3 and Table 1, in which they are described as “min-entropy”. Except for the “Most Common Value” estimation, the M4A files format showed better entropy than the WAV format. This result was rather different from what was expected, because it was presumed that the compressing process would eliminate noises to some extent. However, the compressed works were favorable in terms of entropy estimation, as they increased the randomness of the original signal.

### 4.3. Randomness Estimation According to Channel

To verify the assumption that more randomness may be obtained by using multiple channels, a signal-collecting method was devised. The audio file generation process was exactly the same, except that the channels were changed at every five seconds. Effects by change duration will be analyzed in the following section. In the experiment, four channels were used: 91.3, 101.1, 102.3, and 107.7 MHz, which are all quite strong in Asan, South Korea.

**Figure 4:**
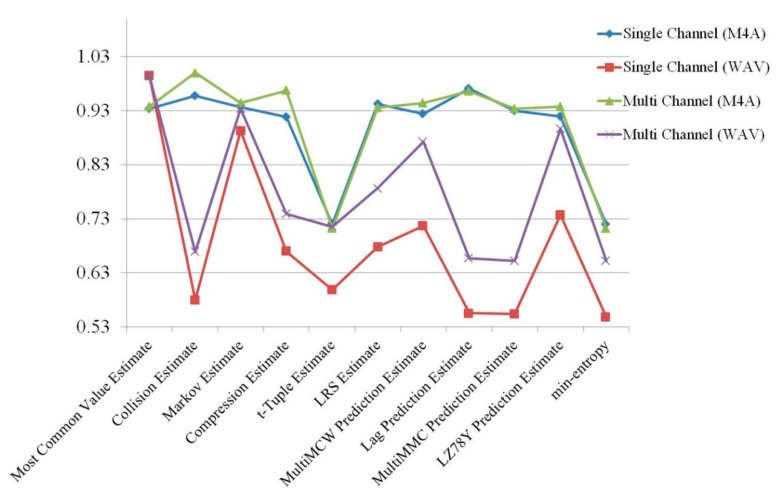
Randomness estimation results comparing single and multiple channels.

Figure 4 shows the results of the randomness estimation for a single channel and multiple channels. For each audio format, five tests were carried out, and the average of the results was used. Even though the M4A file format still had higher entropy than the WAV file format, the entropy of the WAV format increased more than it did for the M4A format. This means that the use of multiple channels enhances randomness better than using single channels.

### 4.4. Randomness Estimation According to Delay

**Figure 5:**
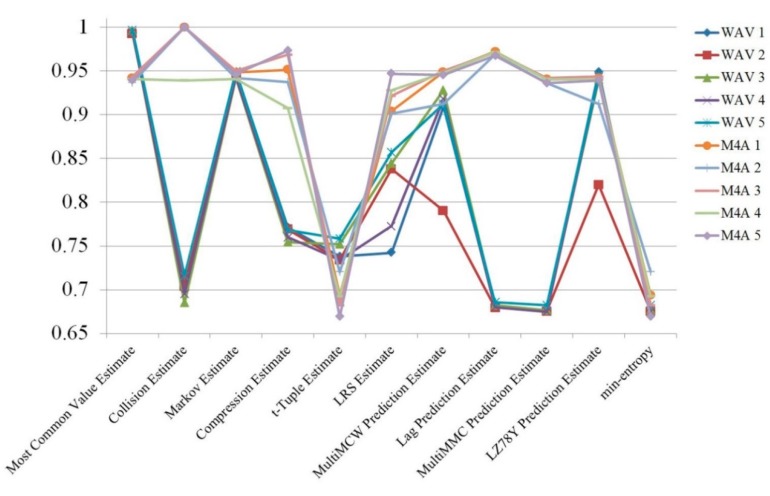
Randomness estimation results of radio signals collected with no delay.

**Figure 6:**
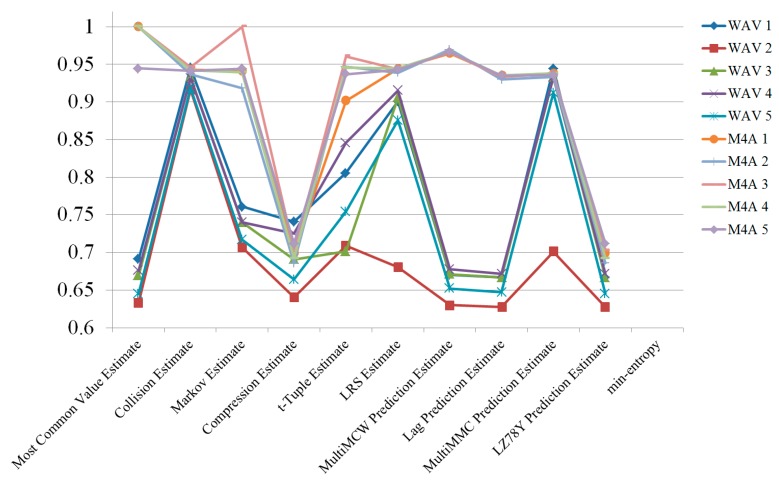
Randomness estimation results of radio signals collected with 1 s delay.

**Figure 7:**
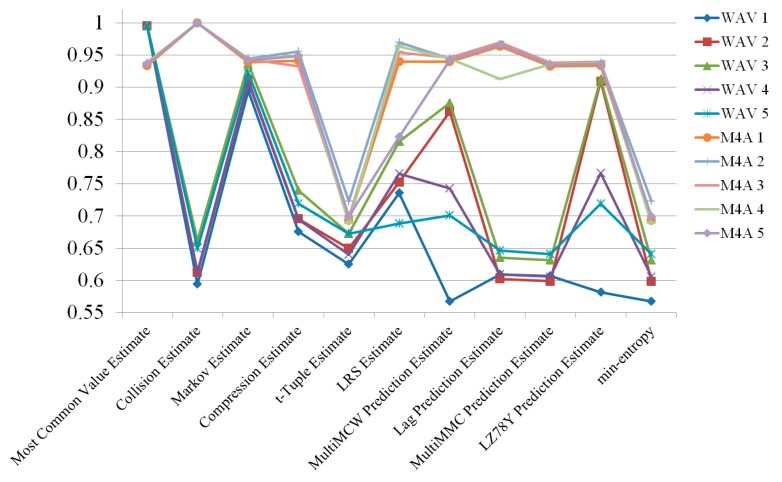
Randomness estimation results of radio signals collected with 2 s delay.

**Figure 8:**
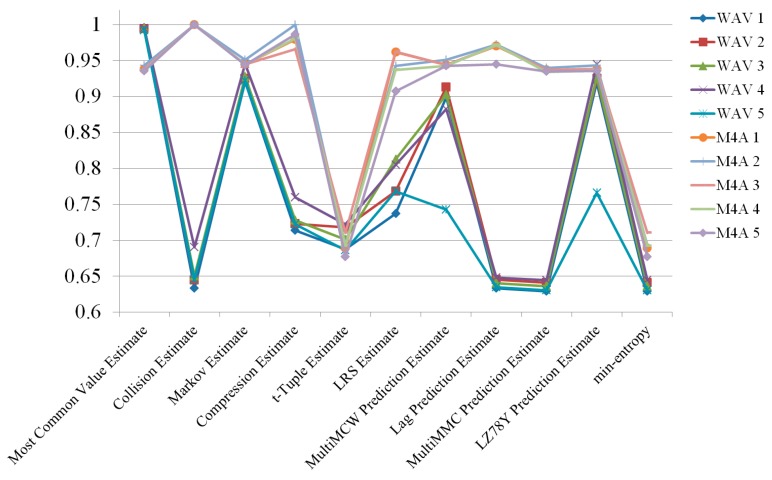
Randomness estimation results of the radio signals collected with 5 s delay.

**Figure 9:**
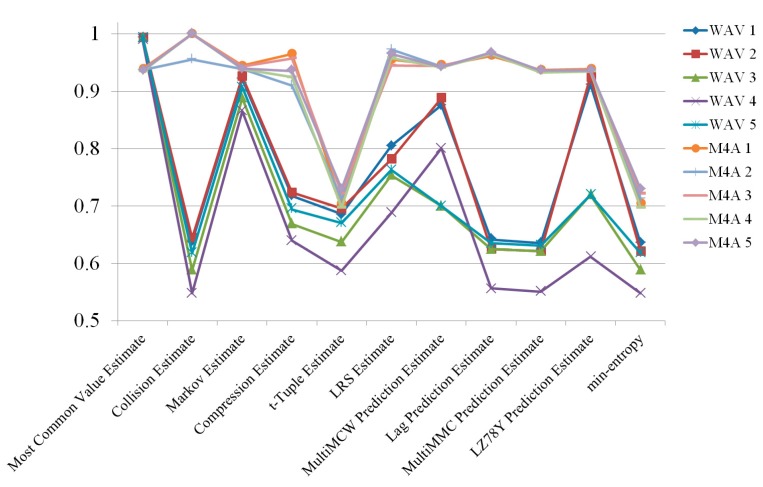
Randomness estimation results of the radio signals collected with 10 s delay.

As final tweaks, randomness was compared based on how multiple channels were changed. In the previous section, we witnessed how the use of multiple channel can improve the entropy of random numbers. With a focus on the delay before changing to a different channel, five channel-changing delays were tested: 1, 2, 5, and 10 s, as well as no delay. No delay means that the four channels were programmed to alternate as quickly as possible, mixing channels at the fasted speed. In each scenario with a specific duration, channel changes occurred after the specified duration. The estimated results are shown in Figure 5, Figure 6, Figure 7, Figure 8 and Figure 9.

**Figure 10:**
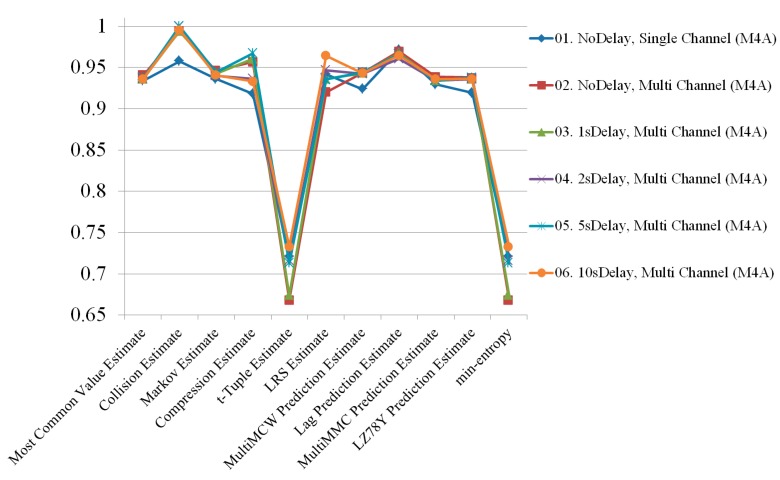
Randomness comparison results of the M4A file format generated from radio signals collected under all conditions.

**Figure 11:**
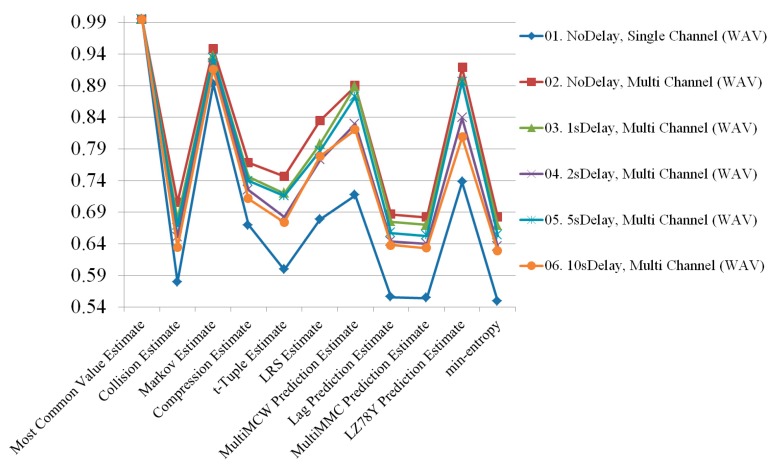
Randomness comparison results of the WAV file format generated from radio signals collected under all conditions.

**Figure 12:**
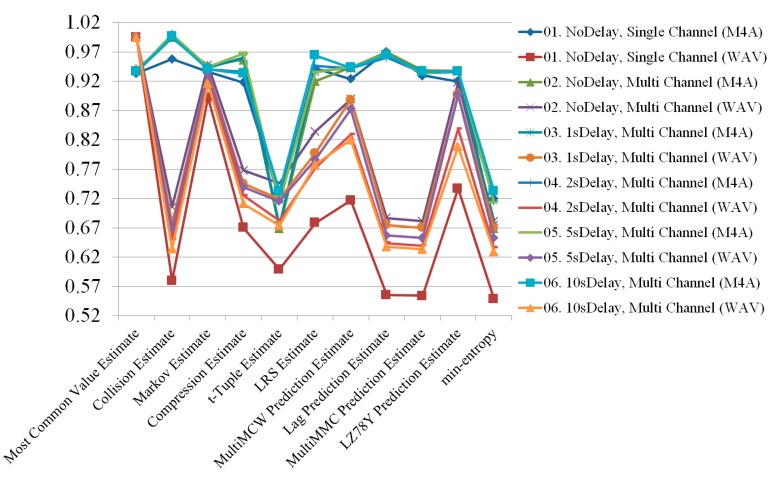
Randomness comparison results of radio signals collected under all conditions.

According to the experimental results, the M4A file format always had higher entropy than the WAV file format. The channel mixing benefits could not overcome the file format difference in terms of quality of entropy. To demonstrate how delay affected the entropy, the two file formats are depicted separately in Figure 10 and Figure 11. The M4A file format had no significant change in entropy, even when the delay was changed. However, the WAV file type had varying entropies of 0.681814, 0.669576, 0.635659, 0.652703, and 0.62834 for no delay and 1, 2, 5, and 10 s delay, respectively. The entropy at all lengths of delay was lower than the entropy with no delay. Results of the radio signals collected under all conditions are shown in Figure 12.

As a result, the entropy for the radio signal using multiple channels with a 10-s delay in the M4A file format had the highest value at 0.733125. On the other hand, the entropy with no delay using multiple channels had the lowest value at 0.668624. For the WAV file, the entropy for the radio signal using multiple channels with no delay was the highest at 0.681814, while for the single channel it was the lowest at 0.548512. Specifically, when collecting radio signals while changing from a single channel to multiple channels, the entropy increased by more than 124%. This means that more random numbers could be collected. Entropies of the M4A and WAV file formats generated from radio signals collected under all conditions are shown in Table 2 and Table 3, respectively.

The best entropy and min-entropy estimated in this study were 1 and 0.768927, respectively. Those of random number generators in previous studies ranged from 0.17 to 0.62, which were lower than this study’s random number generator. Therefore, the proposed generator increased the entropy by at least 118%, and up to 431% compared with previous generators. Comparison results are shown in Table 4.

Radio signals can be used as a good source of noise for random number generators. In addition, rather than simply using the original signal captured from the antenna, the entropy can be increased by mixing three methods: file format, multiple channels, and delay. M4A showed a better entropy than WAV. Since the compression rate of M4A is higher than WAV, other formats with better compression rates could be used as input file formats. Also, multiple instead of single channels are preferable for better entropy. In regards to delay, the shorter the delay, the better entropy. In summary, it was determined that an IoT device can achieve increased entropy when generating random numbers using an audio format with a high compression rate and multiple channels that can be mixed with a short delay.

Please note that we assumed some capabilities of IoT devices. Since an FM modulator is very small and cheap, the use of multiple channels with a short delay is not hard to achieve at all. However, some IoT devices cannot transform the captured signal into audio file formats due to performance and storage limitation. Our study is still meaningful, because even in this scenario IoT devices can choose to mix channels with no delay to increase entropy.

As we continue to depend on mobile and embedded devices for success, secure communication of those devices will be increasingly critical. For this purpose, we suggest that device developers install an FM modulator in each device. With this addition, there can be many applications that utilize the modulator.

## 5. Conclusions

This study proposed a true random number generator using radio signals as a source. Radio signals output from a connected speaker were recorded through an iPhone’s microphone to collect as much noise as possible. The noises were then mingled with radio signals transmitted from the FM radio transmitter, antenna, speaker, and microphone. Comparisons were made based on M4A and WAV file formats using single or multiple channels with various delays. For reliability, one million data were collected to measure randomness using an entropy estimation tool provided by NIST.

Based on the experiments, the M4A file format had higher entropy than the WAV file format. This was because the data compressed to form the M4A enhanced randomness. Moreover, collecting radio signals from multiple channels increased the randomness versus a single channel. Concerning delay, the M4A file format showed no significant change in entropy when delay time was changed. However, when mixing changing channels quickly for the WAV file format, entropy was increased by more than 124%. Compared to previously proposed generators, this study’s generator increased entropy by up to 431%.

The main contribution of this study was the devised method of utilizing FM radio signals for better TRNGs. Since mobile and embedded devices will be located in many different places, signal status cannot always be the same. Additionally, in similar situations, a far better scheme to enhance entropy was proposed in which multiple channels are mixed using a shorter delay. This will help small IoT devices protect their data. Currently, we are performing our study to apply this idea to small IoTs with limited resources.

## Figures and Tables

**Table 1:** Randomness estimation results of 10 radio signals collected from a single channel.

**Estimation Method**		**WAV 1**	**WAV 2**	**WAV 3**	**WAV 4**	**WAV 5**
Running entropic statistic	Most Common Value	0.994655	0.993942	0.994235	0.995983	0.994816
	Collision	0.597521	0.576359	0.543132	0.580566	0.569025
	Markov	0.906099	0.888378	0.866262	0.897765	0.88823
	Compression	0.687897	0.663982	0.635181	0.674413	0.662727
	t-Tuple	0.542449	0.566234	0.616204	0.608734	0.616376
	LRS (Longest Repeated Substring)	0.71991	0.656969	0.662162	0.696862	0.656142
Running predictor	MultiMCW Prediction	0.506145	0.700792	0.790547	0.790547	0.719229
	Lag Prediction	0.552917	0.550647	0.532003	0.547817	0.548995
	MultiMMC Prediction	0.564615	0.548524	0.527592	0.545374	0.545893
	LZ78Y Prediction	0.516184	0.719229	0.820091	0.820091	0.742612
Min-entropy		0.506145	0.548524	0.527593	0.545374	0.545893
**Estimation method**		**WAV 1**	**WAV 2**	**WAV 3**	**WAV 4**	**WAV 5**
Running entropic statistic	Most Common Value	0.932445	0.936669	0.930464	0.933158	0.93275
	Collision	1	0.928538	1	0.966577	0.939304
	Markov	0.937034	0.939917	0.935848	0.936142	0.935433
	Compression	0.935931	0.919182	0.93223	0.919849	0.908234
	t-Tuple	0.717329	0.706209	0.712956	0.710181	0.715842
	LRS	0.946576	0.945553	0.919565	0.933753	0.909221
Running predictor	MultiMCW Prediction	0.938216	0.875879	0.936594	0.939527	0.938808
	Lag Prediction	0.976037	0.971965	0.966401	0.97011	0.966209
	MultiMMC Prediction	0.930236	0.935518	0.929279	0.931495	0.929996
	LZ78Y Prediction	0.932638	0.875879	0.930558	0.93323	0.932927
Min-entropy		0.717329	0.706209	0.712956	0.710181	0.715842

**Table 2:** Entropy comparison results of the m4a file format generated from radio signals collected under all conditions.

Estimation Method		01. No Delay, Single Channel	02. No Delay, Multi-Channel	03. 1 s Delay, Multi-Channel	04. 2 s Delay, Multi-Channel	05. 5 s Delay, Multi-Channel	06. 10 s Delay, Multi-Channel
Running entropic statistic	Most Common Value	0.933612	0.940225	0.936399	0.935789	0.937082	0.936511
	Collision	0.957622	0.99393	0.994472	1	1	0.995561
	Markov	0.936531	0.945996	0.942629	0.940191	0.944155	0.94031
	Compression	0.91804	0.956772	0.960309	0.936181	0.967213	0.933045
	t-Tuple	0.720112	0.668618	0.673698	0.715713	0.712278	0.733125
	LRS	0.941863	0.919989	0.936585	0.946401	0.935651	0.964169
Running predictor	MultiMCW Prediction	0.923763	0.943768	0.942848	0.942247	0.944469	0.943022
	Lag Prediction	0.970942	0.969445	0.966947	0.960397	0.966124	0.964818
	MultiMMC Prediction	0.929884	0.938918	0.933626	0.934693	0.93386	0.935826
	LZ78Y Prediction	0.919499	0.937869	0.936445	0.935851	0.937886	0.936582
Min-entropy		0.720112	0.668624	0.673698	0.715713	0.712278	0.733125

**Table 3:** Entropy comparison result of the WAV file format generated from radio signals collected under all conditions.

Estimation Method		01. No Delay, Single Channel	02. No Delay, Multi-Channel	03. 1 s Delay, Multi-Channel	04. 2 s Delay, Multi-Channel	05. 5 s Delay, Multi-Channel	06. 10 s Delay, Multi-Channel
Running entropic statistic	Most Common Value	0.994507	0.994286	0.994272	0.99447	0.994504	0.994346
	Collision	0.57961	0.70599	0.678468	0.650522	0.668715	0.634806
	Markov	0.892691	0.947569	0.936113	0.925193	0.933679	0.916204
	Compression	0.67003	0.768081	0.745555	0.725115	0.739648	0.711152
	t-Tuple	0.599286	0.746326	0.719644	0.682284	0.715379	0.674293
	LRS	0.678239	0.834761	0.979362	0.772139	0.786462	0.778198
Running predictor	MultiMCW Prediction	0.716853	0.889148	0.88895	0.82928	0.872635	0.820236
	Lag Prediction	0.555752	0.686498	0.674593	0.643926	0.657361	0.63808
	MultiMMC Prediction	0.554359	0.681814	0.669806	0.639643	0.652703	0.633356
	LZ78Y Prediction	0.737101	0.918076	0.896527	0.838326	0.896264	0.808788
Min-entropy		0.548512	0.681814	0.669576	0.635659	0.652703	0.62834

**Table 4:** Comparison with existing random number generators.

Entropy Source	Estimated Entropy	Sample Size	Entropy Source	Reference
Wireless (LQI)	0.47	8 bits	Wireless (LQI)	Hennebert et al. [7]
Accelerometer X	0.22	9 bits	Accelerometer X	Hennebert et al. [8]
Accelerometer Y	0.42	9 bits	Accelerometer Y	
Accelerometer Z	0.36	9 bits	Accelerometer Z	
Vibration sensor	0.17	16 bits	Vibration sensor	
Magnetic sensor	0.62	16 bits	Magnetic sensor	
GTX 690	0.50	4 bits	GTX 690	Yoo et al. [9]
GTX 780	0.60	4 bits	GTX 780	
FM radio	Best entropy: 1.0 Best min-entropy: 0.733125	1 bit	FM radio signal	Our result
